# 6,8-Di­chloro-4-oxochromene-3-carbalde­hyde

**DOI:** 10.1107/S1600536813022228

**Published:** 2013-08-14

**Authors:** Yoshinobu Ishikawa, Yuya Motohashi

**Affiliations:** a School of Pharmaceutical Sciences, University of Shizuoka, 52-1 Yada, Suruga-ku, Shizuoka 422-8526, Japan

## Abstract

The asymmetric unit of the title compound, C_10_H_4_Cl_2_O_3_, contain two essentially planar independent mol­ecules (mean atomic deviations from the corresponding least-square planes are 0.041 and 0.045 Å for mol­ecules 1 and 2, respectively). In the crystal, mol­ecules are linked through a pair of halogen bonds [Cl⋯O separations are 3.044 (5) and 3.033 (6) Å, C—Cl⋯O angles are 160.4 (3) and 162.8 (3)°, and C=O⋯Cl angles are 138.7 (4) and 139.6 (4)°, respectively, in mol­ecules 1 and 2] and C—H⋯O hydrogen bonds into slightly folded bands [the dihedral angle between the planes of neighboring mol­ecules is 8.6 (2)°] along the *c-*axis direction.

## Related literature

For the biological activity of the title and related compounds, see: Shim *et al.* (2003 ▶); Kawase *et al.* (2007 ▶); Dückert *et al.* (2012 ▶). For related structures, see: Ishikawa *et al.* (2013*a* ▶,*b* ▶). For halogen bonding, see: Auffinger *et al.* (2004 ▶); Metrangolo *et al.* (2005 ▶); Wilcken *et al.* (2013 ▶).
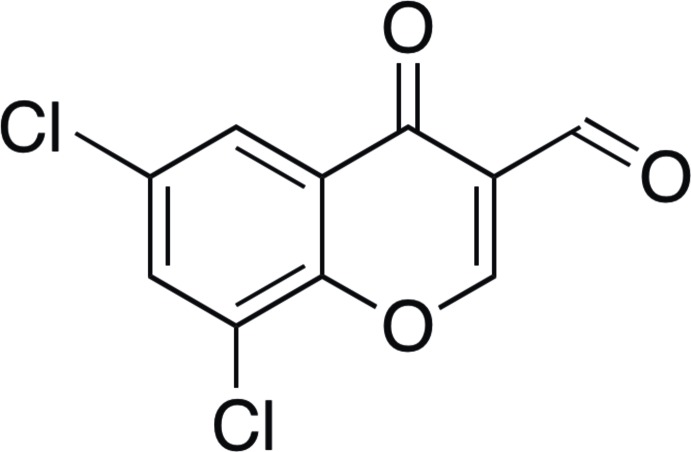


## Experimental

### 

#### Crystal data

C_10_H_4_Cl_2_O_3_
*M* *_r_* = 243.05
Triclinic, 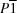

*a* = 8.288 (8) Å
*b* = 8.325 (7) Å
*c* = 13.706 (7) Å
α = 96.55 (6)°
β = 92.23 (7)°
γ = 101.98 (7)°
*V* = 917.2 (13) Å^3^
*Z* = 4
Mo *K*α radiation
μ = 0.68 mm^−1^
*T* = 100 K
0.42 × 0.22 × 0.08 mm

#### Data collection

Rigaku AFC-7R diffractometer
Absorption correction: ψ scan (North *et al.*, 1968 ▶) *T*_min_ = 0.841, *T*_max_ = 0.947
5130 measured reflections
4203 independent reflections
2596 reflections with *F* ^2^ > 2σ(*F* ^2^)
*R*_int_ = 0.057
3 standard reflections every 150 reflections intensity decay: 4.9%

#### Refinement

*R*[*F* ^2^ > 2σ(*F* ^2^)] = 0.076
*wR*(*F* ^2^) = 0.212
*S* = 1.10
4203 reflections
271 parameters
H-atom parameters constrained
Δρ_max_ = 0.70 e Å^−3^
Δρ_min_ = −0.79 e Å^−3^

### 

Data collection: *WinAFC* (Rigaku, 1999 ▶); cell refinement: *WinAFC*; data reduction: *WinAFC*; program(s) used to solve structure: *SHELXS97* (Sheldrick, 2008 ▶); program(s) used to refine structure: *SHELXL97* (Sheldrick, 2008 ▶); molecular graphics: *CrystalStructure* (Rigaku, 2010 ▶); software used to prepare material for publication: *CrystalStructure*.

## Supplementary Material

Crystal structure: contains datablock(s) General, I. DOI: 10.1107/S1600536813022228/ld2111sup1.cif

Structure factors: contains datablock(s) I. DOI: 10.1107/S1600536813022228/ld2111Isup2.hkl

Supplementary material file. DOI: 10.1107/S1600536813022228/ld2111Isup3.cml

Additional supplementary materials:  crystallographic information; 3D view; checkCIF report

## Figures and Tables

**Table 1:** Hydrogen-bond geometry (Å, °)

*D*—H⋯*A*	*D*—H	H⋯*A*	*D*⋯*A*	*D*—H⋯*A*
C4*b* ^i^—H2*b* ^i^⋯O2*a*	0.95	2.35	3.246 (8)	157
C4*a*—H2*a*⋯O2*b* ^i^	0.95	2.35	3.259 (8)	160

Symmetry code: (i) 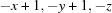.

**Fig. 1.**
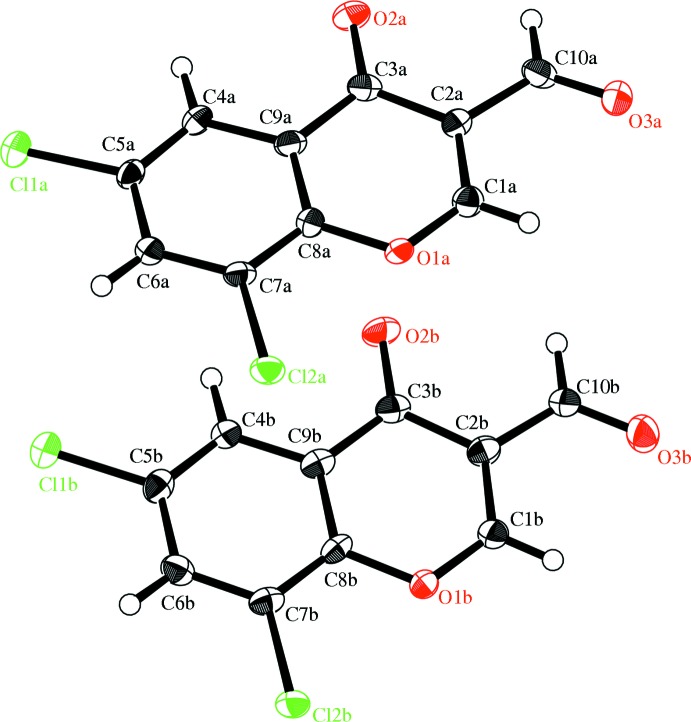
The molecular structure of the title compound, with displacement ellipsoids drawn at the 50% probability level. Hydrogen atoms are shown as small spheres of arbitrary radius.

**Fig. 2.**
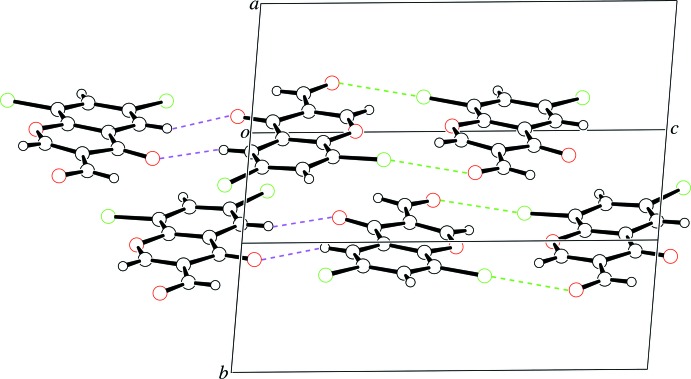
A view of the intermolecular interactions of the title compound, represented as dashed green lines for Cl···O and dashed magenta lines for C–H···O interactions.

## supplementary crystallographic information

### 1. Comment

6,8-Dichloro-3-formylchromone shows many biological functions such as protein tyrosine phosphatase inhibitory (Shim *et al.* 2003), tumor cell-cytotoxic, anti-HIV, anti-*Helicobacter pylori*, and urease inhibitory activities (Kawase *et al.* 2007). In addition, it is used as a starting material for the synthesis of biologically relevant molecules (Dückert *et al.* 2012).

The title compound, C_10_H_4_Cl_2_O_3_, crystallizes with two independent molecules in the asymmetric unit (Fig. 1). The mean deviations from the least-square planes for all atoms of molecule 1 and 2 are 0.0410 Å and 0.0449 Å, respectively. In addition, the largest deviations of molecule 1 and 2 are 0.1512 (17) Å for Cl1a and -0.0973 Å for H4b, respectively. This means that all atoms of each molecule are essentially coplanar.

In the crystal, the molecules 1 and 2 are linked to each other through intermolecular interactions of the Cl atoms at the 8-position with the O atoms of the formyl groups [Cl2a···O3b^i^; 3.033 (6) Å, Cl2b^i^···O3a; 3.044 (5) Å, C7a–Cl2a···O3b^i^ = 160.4 (3)°, C7b^i^–Cl2b^i^···O3a =162.8 (3)°, C10*a*–O3a···Cl2b^i^ = 138.7 (4)°, C10*b*^i^–O3b^i^···Cl2a = 139.6 (4)° (i): -*x* + 1, -*y* + 1, -*z* + 1], and the carbonyl O atoms at the 4-position with the C–H atoms at the 5-position. The short contacts and the geometries involved in the Cl atoms fall into halogen bonding (Auffinger *et al.* 2004). Due to these halogen and hydrogen bonds, the molecules form wavy bands along *c* axis, as shown in Fig. 2.

Halogen bonds have been found to occur in organic, inorganic, and biological systems, and have recently attracted much attention in medicinal chemistry, chemical biology, and supramolecular chemistry (Auffinger *et al.* 2004, Metrangolo *et al.* 2005, Wilcken *et al.* 2013). Our analysis suggests that the strong inhibitory activity of the title compound against urease may be attributable to the halogen bond observed in the crystal, because 3-formylchromones without any halogen atom at the 8-position in the literature do not show the urease inhibitory activity (Kawase *et al.* 2007).

### 2. Experimental

Single crystals suitable for X-ray diffraction were obtained by slow evaporation of a 2-butanone solution of commercially available 6,8-dichloro-3-formylchromone at room temperature.

### 3. Refinement

The C(sp^2^)-bound hydrogen atoms were placed in geometrical positions [C–H 0.95 Å, *U*_iso_(H) = 1.2*U*_eq_(C)], and refined using a riding model.

### Crystal data

**Table:**

C_10_H_4_Cl_2_O_3_	*Z* = 4
*M**_r_* = 243.05	*F*(000) = 488.00
Triclinic, *P*1	*D*_x_ = 1.760 Mg m^−^^3^
Hall symbol: -P 1	Mo *K*α radiation, λ = 0.71069 Å
*a* = 8.288 (8) Å	Cell parameters from 23 reflections
*b* = 8.325 (7) Å	θ = 15.2–17.4°
*c* = 13.706 (7) Å	µ = 0.68 mm^−^^1^
α = 96.55 (6)°	*T* = 100 K
β = 92.23 (7)°	Prismatic, colourless
γ = 101.98 (7)°	0.42 × 0.22 × 0.08 mm
*V* = 917.2 (13) Å^3^	

### Data collection

**Table:**

Rigaku AFC-7R diffractometer	*R*_int_ = 0.057
ω–2θ scans	θ_max_ = 27.5°
Absorption correction: ψ scan (North *et al.*, 1968)	*h* = −10→6
*T*_min_ = 0.841, *T*_max_ = 0.947	*k* = −10→10
5130 measured reflections	*l* = −17→17
4203 independent reflections	3 standard reflections every 150 reflections
2596 reflections with *F*^2^ > 2σ(*F*^2^)	intensity decay: 4.9%

### Refinement

**Table:**

Refinement on *F*^2^	Secondary atom site location: difference Fourier map
*R*[*F*^2^ > 2σ(*F*^2^)] = 0.076	Hydrogen site location: inferred from neighbouring sites
*wR*(*F*^2^) = 0.212	H-atom parameters constrained
*S* = 1.10	*w* = 1/[σ^2^(*F*_o_^2^) + (0.0531*P*)^2^ + 6.620*P*] where *P* = (*F*_o_^2^ + 2*F*_c_^2^)/3
4203 reflections	(Δ/σ)_max_ < 0.001
271 parameters	Δρ_max_ = 0.70 e Å^−^^3^
0 restraints	Δρ_min_ = −0.79 e Å^−^^3^
Primary atom site location: structure-invariant direct methods	

### Special details

**Table:**

Refinement. Refinement was performed using all reflections. The weighted *R*-factor (*wR*) and goodness of fit (*S*) are based on *F*^2^. *R*-factor (gt) are based on *F*. The threshold expression of *F*^2^ > 2.0 σ(*F*^2^) is used only for calculating *R*-factor (gt).

### Fractional atomic coordinates and isotropic or equivalent isotropic displacement parameters (Å^2^)

**Table:**

	*x*	*y*	*z*	*U*_iso_*/*U*_eq_	
Cl1a	0.9284 (2)	0.7180 (2)	−0.05206 (12)	0.0275 (4)	
Cl2a	0.8997 (2)	0.60177 (19)	0.32529 (11)	0.0243 (4)	
Cl1b	0.8654 (3)	1.0637 (2)	0.20783 (12)	0.0293 (4)	
Cl2b	0.7665 (2)	1.01839 (19)	0.59010 (11)	0.0246 (4)	
O1a	0.6255 (6)	0.3339 (5)	0.2521 (3)	0.0218 (10)	
O2a	0.4506 (6)	0.1743 (6)	−0.0336 (4)	0.0277 (11)	
O3a	0.2398 (7)	−0.0767 (6)	0.1869 (4)	0.0319 (12)	
O1b	0.4940 (6)	0.7493 (6)	0.5162 (3)	0.0219 (10)	
O2b	0.3559 (6)	0.5491 (6)	0.2305 (3)	0.0291 (11)	
O3b	0.1021 (7)	0.3424 (6)	0.4522 (4)	0.0326 (12)	
C1a	0.4980 (8)	0.2042 (8)	0.2288 (5)	0.0221 (13)	
C2a	0.4342 (8)	0.1454 (8)	0.1358 (5)	0.0197 (13)	
C3a	0.5023 (8)	0.2255 (8)	0.0520 (5)	0.0217 (13)	
C4a	0.7126 (9)	0.4638 (8)	0.0063 (5)	0.0242 (14)	
C5a	0.8382 (8)	0.5956 (8)	0.0344 (5)	0.0201 (13)	
C6a	0.9020 (8)	0.6399 (8)	0.1321 (5)	0.0202 (13)	
C7a	0.8290 (8)	0.5497 (8)	0.2026 (5)	0.0197 (13)	
C8a	0.6945 (8)	0.4171 (8)	0.1770 (5)	0.0202 (13)	
C9a	0.6370 (9)	0.3695 (8)	0.0784 (5)	0.0207 (13)	
C10a	0.2954 (9)	−0.0026 (8)	0.1204 (5)	0.0250 (14)	
C1b	0.3630 (8)	0.6197 (8)	0.4918 (5)	0.0220 (13)	
C2b	0.3130 (8)	0.5479 (8)	0.4003 (5)	0.0217 (13)	
C3b	0.3958 (8)	0.6127 (8)	0.3161 (5)	0.0214 (13)	
C4b	0.6216 (8)	0.8361 (8)	0.2689 (5)	0.0220 (13)	
C5b	0.7526 (8)	0.9654 (8)	0.2961 (5)	0.0227 (14)	
C6b	0.8016 (9)	1.0234 (8)	0.3958 (5)	0.0234 (14)	
C7b	0.7127 (8)	0.9495 (8)	0.4674 (5)	0.0217 (13)	
C8b	0.5792 (8)	0.8170 (8)	0.4411 (5)	0.0188 (13)	
C9b	0.5333 (8)	0.7557 (8)	0.3414 (5)	0.0190 (13)	
C10b	0.1768 (9)	0.4034 (8)	0.3852 (5)	0.0238 (14)	
H1a	0.4491	0.1499	0.2811	0.0265*	
H2a	0.6755	0.4349	−0.0611	0.0290*	
H3a	0.9933	0.7302	0.1493	0.0243*	
H4a	0.2484	−0.0406	0.0555	0.0300*	
H1b	0.3024	0.5769	0.5440	0.0264*	
H2b	0.5904	0.8006	0.2012	0.0264*	
H3b	0.8945	1.1120	0.4131	0.0281*	
H4b	0.1440	0.3536	0.3196	0.0286*	

### Atomic displacement parameters (Å^2^)

**Table:**

	*U*^11^	*U*^22^	*U*^33^	*U*^12^	*U*^13^	*U*^23^
Cl1a	0.0338 (9)	0.0277 (9)	0.0216 (8)	0.0044 (7)	0.0053 (7)	0.0088 (6)
Cl2a	0.0312 (9)	0.0231 (8)	0.0166 (7)	0.0037 (7)	−0.0028 (6)	−0.0007 (6)
Cl1b	0.0345 (10)	0.0287 (9)	0.0246 (8)	0.0034 (8)	0.0082 (7)	0.0071 (7)
Cl2b	0.0311 (9)	0.0232 (8)	0.0171 (7)	0.0031 (7)	−0.0008 (6)	−0.0016 (6)
O1a	0.034 (3)	0.019 (3)	0.012 (2)	0.0034 (19)	0.0043 (18)	0.0014 (16)
O2a	0.035 (3)	0.032 (3)	0.014 (3)	0.005 (3)	−0.0023 (19)	−0.0005 (18)
O3a	0.037 (3)	0.030 (3)	0.023 (3)	−0.005 (3)	0.006 (2)	0.002 (2)
O1b	0.029 (3)	0.021 (3)	0.014 (2)	0.0013 (19)	0.0014 (18)	0.0028 (17)
O2b	0.035 (3)	0.035 (3)	0.014 (3)	0.003 (3)	−0.0017 (19)	−0.0023 (19)
O3b	0.036 (3)	0.032 (3)	0.025 (3)	−0.007 (3)	−0.002 (3)	0.005 (2)
C1a	0.025 (4)	0.022 (4)	0.020 (3)	0.006 (3)	0.001 (3)	0.004 (3)
C2a	0.020 (4)	0.021 (3)	0.018 (3)	0.005 (3)	0.003 (3)	0.001 (3)
C3a	0.026 (4)	0.020 (3)	0.019 (3)	0.007 (3)	−0.001 (3)	−0.001 (3)
C4a	0.033 (4)	0.025 (4)	0.013 (3)	0.003 (3)	0.003 (3)	0.004 (3)
C5a	0.028 (4)	0.019 (3)	0.017 (3)	0.010 (3)	0.007 (3)	0.006 (3)
C6a	0.025 (4)	0.017 (3)	0.019 (3)	0.004 (3)	0.003 (3)	0.002 (3)
C7a	0.025 (4)	0.020 (3)	0.014 (3)	0.006 (3)	−0.001 (3)	−0.001 (3)
C8a	0.024 (4)	0.019 (3)	0.017 (3)	0.004 (3)	0.002 (3)	0.004 (3)
C9a	0.030 (4)	0.018 (3)	0.015 (3)	0.009 (3)	0.002 (3)	0.001 (3)
C10a	0.030 (4)	0.019 (3)	0.024 (4)	0.004 (3)	−0.002 (3)	−0.001 (3)
C1b	0.027 (4)	0.019 (3)	0.021 (3)	0.006 (3)	0.004 (3)	0.002 (3)
C2b	0.023 (4)	0.025 (4)	0.018 (3)	0.007 (3)	−0.001 (3)	0.002 (3)
C3b	0.028 (4)	0.022 (4)	0.017 (3)	0.011 (3)	−0.000 (3)	0.002 (3)
C4b	0.027 (4)	0.021 (3)	0.018 (3)	0.004 (3)	0.004 (3)	0.002 (3)
C5b	0.028 (4)	0.023 (4)	0.022 (4)	0.012 (3)	0.006 (3)	0.008 (3)
C6b	0.027 (4)	0.020 (4)	0.022 (4)	0.004 (3)	−0.003 (3)	0.001 (3)
C7b	0.026 (4)	0.024 (4)	0.016 (3)	0.010 (3)	−0.000 (3)	0.001 (3)
C8b	0.023 (4)	0.024 (4)	0.014 (3)	0.013 (3)	0.005 (3)	0.006 (3)
C9b	0.020 (3)	0.023 (3)	0.017 (3)	0.013 (3)	0.000 (3)	0.003 (3)
C10b	0.031 (4)	0.019 (3)	0.020 (3)	0.004 (3)	0.004 (3)	0.000 (3)

### Geometric parameters (Å, º)

**Table:**

Cl1a—C5a	1.740 (7)	C7a—C8a	1.398 (8)
Cl2a—C7a	1.735 (6)	C8a—C9a	1.401 (9)
Cl1b—C5b	1.733 (7)	C1b—C2b	1.338 (9)
Cl2b—C7b	1.723 (6)	C2b—C3b	1.464 (9)
O1a—C1a	1.344 (7)	C2b—C10b	1.458 (9)
O1a—C8a	1.381 (8)	C3b—C9b	1.465 (8)
O2a—C3a	1.229 (8)	C4b—C5b	1.367 (9)
O3a—C10a	1.210 (9)	C4b—C9b	1.405 (9)
O1b—C1b	1.363 (7)	C5b—C6b	1.411 (9)
O1b—C8b	1.379 (8)	C6b—C7b	1.376 (10)
O2b—C3b	1.233 (7)	C7b—C8b	1.393 (8)
O3b—C10b	1.227 (9)	C8b—C9b	1.412 (8)
C1a—C2a	1.356 (9)	C1a—H1a	0.950
C2a—C3a	1.467 (9)	C4a—H2a	0.950
C2a—C10a	1.490 (9)	C6a—H3a	0.950
C3a—C9a	1.459 (9)	C10a—H4a	0.950
C4a—C5a	1.355 (9)	C1b—H1b	0.950
C4a—C9a	1.416 (9)	C4b—H2b	0.950
C5a—C6a	1.401 (9)	C6b—H3b	0.950
C6a—C7a	1.373 (9)	C10b—H4b	0.950
			
C1a—O1a—C8a	118.3 (5)	C5b—C4b—C9b	119.9 (6)
C1b—O1b—C8b	118.1 (5)	Cl1b—C5b—C4b	120.6 (5)
O1a—C1a—C2a	124.6 (6)	Cl1b—C5b—C6b	117.6 (5)
C1a—C2a—C3a	120.4 (6)	C4b—C5b—C6b	121.9 (6)
C1a—C2a—C10a	118.9 (6)	C5b—C6b—C7b	118.9 (6)
C3a—C2a—C10a	120.7 (6)	Cl2b—C7b—C6b	120.4 (5)
O2a—C3a—C2a	122.6 (6)	Cl2b—C7b—C8b	119.6 (5)
O2a—C3a—C9a	122.9 (6)	C6b—C7b—C8b	120.0 (6)
C2a—C3a—C9a	114.5 (5)	O1b—C8b—C7b	117.3 (5)
C5a—C4a—C9a	119.4 (6)	O1b—C8b—C9b	121.6 (5)
Cl1a—C5a—C4a	120.3 (5)	C7b—C8b—C9b	121.1 (6)
Cl1a—C5a—C6a	117.0 (5)	C3b—C9b—C4b	121.9 (6)
C4a—C5a—C6a	122.7 (6)	C3b—C9b—C8b	119.8 (6)
C5a—C6a—C7a	118.3 (6)	C4b—C9b—C8b	118.2 (5)
Cl2a—C7a—C6a	120.3 (5)	O3b—C10b—C2b	123.8 (6)
Cl2a—C7a—C8a	119.0 (5)	O1a—C1a—H1a	117.700
C6a—C7a—C8a	120.6 (6)	C2a—C1a—H1a	117.702
O1a—C8a—C7a	117.4 (5)	C5a—C4a—H2a	120.314
O1a—C8a—C9a	122.2 (5)	C9a—C4a—H2a	120.307
C7a—C8a—C9a	120.4 (6)	C5a—C6a—H3a	120.850
C3a—C9a—C4a	121.6 (6)	C7a—C6a—H3a	120.844
C3a—C9a—C8a	119.9 (6)	O3a—C10a—H4a	118.481
C4a—C9a—C8a	118.5 (6)	C2a—C10a—H4a	118.489
O3a—C10a—C2a	123.0 (6)	O1b—C1b—H1b	117.382
O1b—C1b—C2b	125.2 (6)	C2b—C1b—H1b	117.374
C1b—C2b—C3b	120.2 (6)	C5b—C4b—H2b	120.070
C1b—C2b—C10b	119.4 (6)	C9b—C4b—H2b	120.068
C3b—C2b—C10b	120.3 (6)	C5b—C6b—H3b	120.568
O2b—C3b—C2b	122.6 (6)	C7b—C6b—H3b	120.567
O2b—C3b—C9b	122.5 (6)	O3b—C10b—H4b	118.104
C2b—C3b—C9b	114.9 (5)	C2b—C10b—H4b	118.102
			
C1a—O1a—C8a—C7a	−178.8 (6)	O1a—C8a—C9a—C3a	−1.4 (10)
C1a—O1a—C8a—C9a	−0.7 (9)	O1a—C8a—C9a—C4a	178.7 (6)
C8a—O1a—C1a—C2a	1.9 (10)	C7a—C8a—C9a—C3a	176.7 (6)
C8a—O1a—C1a—H1a	−178.1	C7a—C8a—C9a—C4a	−3.2 (10)
C1b—O1b—C8b—C7b	180.0 (6)	O1b—C1b—C2b—C3b	−2.9 (11)
C1b—O1b—C8b—C9b	0.3 (9)	O1b—C1b—C2b—C10b	176.3 (6)
C8b—O1b—C1b—C2b	2.5 (10)	H1b—C1b—C2b—C3b	177.1
C8b—O1b—C1b—H1b	−177.5	H1b—C1b—C2b—C10b	−3.7
O1a—C1a—C2a—C3a	−1.0 (11)	C1b—C2b—C3b—O2b	178.7 (7)
O1a—C1a—C2a—C10a	178.3 (6)	C1b—C2b—C3b—C9b	0.7 (10)
H1a—C1a—C2a—C3a	179.0	C1b—C2b—C10b—O3b	0.7 (11)
H1a—C1a—C2a—C10a	−1.7	C1b—C2b—C10b—H4b	−179.3
C1a—C2a—C3a—O2a	178.4 (7)	C3b—C2b—C10b—O3b	179.9 (7)
C1a—C2a—C3a—C9a	−1.1 (10)	C3b—C2b—C10b—H4b	−0.1
C1a—C2a—C10a—O3a	−2.4 (11)	C10b—C2b—C3b—O2b	−0.4 (11)
C1a—C2a—C10a—H4a	177.6	C10b—C2b—C3b—C9b	−178.5 (6)
C3a—C2a—C10a—O3a	176.9 (7)	O2b—C3b—C9b—C4b	4.2 (11)
C3a—C2a—C10a—H4a	−3.1	O2b—C3b—C9b—C8b	−176.2 (6)
C10a—C2a—C3a—O2a	−0.9 (11)	C2b—C3b—C9b—C4b	−177.7 (6)
C10a—C2a—C3a—C9a	179.7 (6)	C2b—C3b—C9b—C8b	1.8 (9)
O2a—C3a—C9a—C4a	2.6 (11)	C5b—C4b—C9b—C3b	−177.7 (6)
O2a—C3a—C9a—C8a	−177.3 (6)	C5b—C4b—C9b—C8b	2.7 (10)
C2a—C3a—C9a—C4a	−178.0 (6)	C9b—C4b—C5b—Cl1b	178.8 (6)
C2a—C3a—C9a—C8a	2.2 (10)	C9b—C4b—C5b—C6b	−0.9 (11)
C5a—C4a—C9a—C3a	−179.4 (6)	H2b—C4b—C5b—Cl1b	−1.2
C5a—C4a—C9a—C8a	0.5 (11)	H2b—C4b—C5b—C6b	179.1
C9a—C4a—C5a—Cl1a	−177.7 (6)	H2b—C4b—C9b—C3b	2.3
C9a—C4a—C5a—C6a	2.5 (11)	H2b—C4b—C9b—C8b	−177.3
H2a—C4a—C5a—Cl1a	2.3	Cl1b—C5b—C6b—C7b	179.0 (5)
H2a—C4a—C5a—C6a	−177.5	Cl1b—C5b—C6b—H3b	−1.0
H2a—C4a—C9a—C3a	0.6	C4b—C5b—C6b—C7b	−1.3 (11)
H2a—C4a—C9a—C8a	−179.5	C4b—C5b—C6b—H3b	178.6
Cl1a—C5a—C6a—C7a	177.5 (5)	C5b—C6b—C7b—Cl2b	−179.1 (6)
Cl1a—C5a—C6a—H3a	−2.5	C5b—C6b—C7b—C8b	1.6 (11)
C4a—C5a—C6a—C7a	−2.7 (11)	H3b—C6b—C7b—Cl2b	0.9
C4a—C5a—C6a—H3a	177.3	H3b—C6b—C7b—C8b	−178.4
C5a—C6a—C7a—Cl2a	−178.6 (6)	Cl2b—C7b—C8b—O1b	1.3 (9)
C5a—C6a—C7a—C8a	−0.1 (10)	Cl2b—C7b—C8b—C9b	−179.0 (5)
H3a—C6a—C7a—Cl2a	1.4	C6b—C7b—C8b—O1b	−179.3 (6)
H3a—C6a—C7a—C8a	179.9	C6b—C7b—C8b—C9b	0.3 (11)
Cl2a—C7a—C8a—O1a	−0.3 (9)	O1b—C8b—C9b—C3b	−2.4 (10)
Cl2a—C7a—C8a—C9a	−178.5 (5)	O1b—C8b—C9b—C4b	177.2 (6)
C6a—C7a—C8a—O1a	−178.8 (6)	C7b—C8b—C9b—C3b	178.0 (6)
C6a—C7a—C8a—C9a	3.0 (10)	C7b—C8b—C9b—C4b	−2.5 (10)

### Hydrogen-bond geometry (Å, º)

**Table:**

*D*—H···*A*	*D*—H	H···*A*	*D*···*A*	*D*—H···*A*
C4*b*^i^—H2*b*^i^···O2*a*	0.95	2.35	3.246 (8)	157
C4*a*—H2*a*···O2*b*^i^	0.95	2.35	3.259 (8)	160

Symmetry code: (i) −*x*+1, −*y*+1, −*z*.
